# Protective role of remote ischemic conditioning in renal transplantation and partial nephrectomy: A systematic review and meta-analysis of randomized controlled trials

**DOI:** 10.3389/fsurg.2023.1024650

**Published:** 2023-04-05

**Authors:** Wenfu Zhang, Yingting Wu, Mingwang Zeng, Chao Yang, Zhengang Qiu, Rongrong Liu, Lifeng Wang, Maolin Zhong, Qiaoling Chen, Weidong Liang

**Affiliations:** ^1^ The First Clinical Medical College of Gannan Medical University, Ganzhou, China; ^2^ Department of Anesthesia, hospital of Traditional Chinese Medicine of Zhongshan, Zhongshan, China; ^3^ Department of Critical Care Medicine Nursing, the First Affiliated Hospital of Gannan Medical University, Ganzhou, China; ^4^ Department of Oncology, The First Affiliated Hospital of Gannan Medical University, Ganzhou, China; ^5^ Department of Neurology, The First Affiliated Hospital of Gannan Medical University, Ganzhou, China; ^6^ Anesthesia Surgery Center of the First Affiliated Hospital of Gannan Medical University, Ganzhou, China; ^7^ Department of Anesthesiology, The First Affiliated Hospital of Xiamen University, Xiamen, China

**Keywords:** remote ischemic conditioning, ischemia-reperfusion injury, nephroprotection, kidney transplantation, nephrectomy, meta-analysis

## Abstract

**Objective:**

Studies have shown that remote ischemic conditioning (RIC) can effectively attenuate ischemic-reperfusion injury in the heart and brain, but the effect on ischemic-reperfusion injury in patients with kidney transplantation or partial nephrectomy remains controversial. The main objective of this systematic review and meta-analysis was to investigate whether RIC provides renal protection after renal ischemia-reperfusion injury in patients undergoing kidney transplantation or partial nephrectomy.

**Methods:**

A computer-based search was conducted to retrieve relevant publications from the PubMed database, Embase database, Cochrane Library and Web of Science database. We then conducted a systematic review and meta-analysis of randomized controlled trials that met our study inclusion criteria.

**Results:**

Eleven eligible studies included a total of 1,145 patients with kidney transplantation or partial nephrectomy for systematic review and meta-analysis, among whom 576 patients were randomly assigned to the RIC group and the remaining 569 to the control group. The 3-month estimated glomerular filtration rate (eGFR) was improved in the RIC group, which was statistically significant between the two groups on kidney transplantation [*P *< 0.001; mean difference (MD) = 2.74, confidence interval (CI): 1.41 to 4.06; *I*^2 ^= 14%], and the 1- and 2-day postoperative Scr levels in the RIC group decreased, which was statistically significant between the two groups on kidney transplantation (1-day postoperative: *P *< 0.001; MD = 0.10, CI: 0.05 to 0.15, *I*^2 ^= 0; 2-day postoperative: *P *= 0.006; MD = 0.41, CI: 0.12 to 0.70, *I*^2 ^= 0), but at other times, there was no significant difference between the two groups in Scr levels. The incidence of delayed graft function (DGF) decreased, but there was no significant difference (*P *= 0.60; 95% CI: 0.67 to 1.26). There was no significant difference between the two groups in terms of cross-clamp time, cold ischemia time, warm ischemic time, acute rejection (AR), graft loss or length of hospital stay.

**Conclusion:**

Our meta-analysis showed that the effect of remote ischemia conditioning on reducing serum creatinine (Scr) and improving estimate glomerular filtration rate (eGFR) seemed to be very weak, and we did not observe a significant protective effect of RIC on renal ischemic-reperfusion. Due to small sample sizes, more studies using stricter inclusion criteria are needed to elucidate the nephroprotective effect of RIC in renal surgery in the future.

## Introduction

When ischemia occurs in an organ, cell death and tissue damage are inevitable. Although restoring blood flow to ischemic organs can prevent irreversible tissue damage to a certain extent, during reperfusion, reperfusion in turn induces inflammatory responses and tissue damage, leading to organ dysfunction. The cell damage caused by the reperfusion of surviving ischemic tissue is called ischemia-reperfusion (I/R) injury (1). I/R injury can occur spontaneously, as in ischemic stroke, myocardial infarction, transplantation and other types of surgery (2–4). In the kidney, due to factors such as high metabolism and vascular anatomy, the kidney is particularly sensitive to I/R injury. It has been shown that the kidney will cause irreversible damage after tens of minutes of ischemia (5). Therefore, in renal transplantation or partial nephrectomy, I/R injury is an unavoidable event due to occlusion of renal blood vessels, which may lead to delayed graft function (DGF), acute rejection (AR), increased risk of graft loss, etc. (6, 7). To date, various interventions have been evaluated for their effectiveness in reducing the physiological stress of surgery or improving the patient's tolerance to stressors, such as drug intervention, fluid resuscitation, glycaemic control, and maintenance of electrolyte balance, and these have been shown to potentially improve patient outcomes (8). However, due to patient factors, lack of appropriate facilities, and time constraints, these conditions cannot be applied well. Therefore, there is a particular need for a more substantial, low-cost and easy-to-implement intervention to reduce I/R injury in kidney transplantation or partial nephrectomy.

Remote ischemic conditioning (RIC) is a simple and safe noninvasive intervention that applies brief (a few minutes) reversible ischemia and reperfusion to tissues remote from the target organ, which may make target organs more resistant to subsequent ischemia and reperfusion injury (9). At present, the exact mechanism of RIC is not clearly understood. It may involve the modulation of neural, humoral and systemic responses (10). In animal models, RIC has been found to be an effective tool to protect certain organs (11). In most clinical trials, the extremity has many advantages as a distal tissue; ischemic stimulation can be implemented by placing a specially designed blood pressure cuff on the upper or lower extremity. Skeletal muscle is less susceptible to I/R injury than visceral organs (12). Second, the inexpensive economic cost, practical feasibility, and noninvasiveness of RIC make it an emerging and promising method (13). According to different application time points, RIC can be divided into three categories (14): remote ischemic preconditioning (RIPC, induced before target organ ischemia), remote ischemic perconditioning (RIPerC, induced during target organ ischemia but before reperfusion), and remote ischemic postconditioning (RIPostC, induction at the beginning of reperfusion). Currently, many researchers have focused on the role of RIC in the protection of the heart, brain, and liver and have found that RIC seems to be an effective method to reduce I/R injury (15–17). For example, it may decrease long-term clinical events in patients undergoing cardiac surgery or percutaneous coronary intervention (PCI) (18) and reduce hepatic I/R injury after liver resection (19). The nephroprotective effect of RIC has been confirmed in animal experiments, and some studies have demonstrated that RIPC decreases renal I/R injury (20–22). However, in clinical trials, whether RIC alleviates renal I/R injury remains inconclusive. Given the inconsistency and limited sample size of the existing RIC literature, we performed a systematic review and meta-analysis of randomized controlled trials (RCTs) in patients with kidney transplantation or partial nephrectomy to assess the therapeutic effect and safety of RIC in kidney transplantation or partial nephrectomy.

## Methods

### Identification of eligible studies

This meta-analysis was conducted and reported according to the Preferred Reporting Item for Systematic Reviews and Meta-Analyses (PRISMA) guidelines (23). The study was registered with Prospero (registration number: CRD42021291324).

A comprehensive electronic search of PubMed, Embase, Web of Science and the Cochrane library was conducted to identify all eligible studies, last updated 2021.11.03. The search keywords were as follows: remote ischemic preconditioning, remote ischemic perconditioning, remote ischemic postconditioning, RIC, limb RIC or IPC; and kidney transplantation, renal transplantation, renal graft, kidney graft, renal transplant, partial nephrectomy or LPN. Relevant articles and bibliographies were manually searched to avoid omissions. After title screening, we assessed abstracts for relevance and determined whether they were included, excluded, or warranted further evaluation. No restrictions were imposed on publication terms or languages. The search strategies applied in the database were as follows: (1) remote ischemic preconditioning; (2) remote ischemic perconditioning; (3) remote ischemic postconditioning; (4) RIC; (5) limb RIC; (6) IPC; (7): (1) or (2) or (3) or (4) or (5) or (6); (8) kidney transplantation; (9) renal transplantation; (10) renal graft; (11) kidney graft; (12) renal transplant; (13) partial nephrectomy; (14) LPN; (15): (8) or (9) or (10) or (11) or (12) or (13) or (14); (16): (7) and (15).

### Selection criteria

The studies identified in the above databases (PubMed, Embase, Web of Science and Cochrane Library) were screened, and after removal of duplicates, all references were further screened according to the title and abstract of the reference. The screening criteria were as follows: (1) design for clinical RCTs; (2) patients undergoing renal transplantation or partial nephrectomy who received noninvasive RIC (RIPC, RIPerC or RIPostC), regardless of time, pressure or number of cycles. (3) Provide relevant data, such as the estimated glomerular filtration rate (eGFR), serum creatinine (Scr), DGF, AR, graft loss and length of hospital stay. Subsequently, full text manuscripts of eligible studies were reviewed for inclusion. Studies involving combination drugs other than narcotics or pain relievers or other modalities of combination intervention were excluded. For studies with identical or overlapping data from the same author, the most appropriate study with the largest number of cases or the latest publication date was selected. In addition, we excluded retrospective analyses, case reports, conference abstracts, etc. At the above stage, articles were independently evaluated for inclusion by two authors (Wenfu Zhang and Yingting Wu).

### Quality assessment

Reviewers used the Cochrane Collaboration tool for risk of bias assessment (24). For each included study, a judgement of “high”, “unclear” or “low” risk of bias was provided for each of the following areas: adequate random sequence generation; allocation sequence concealment; blinding of results; incomplete outcome data; no selective outcome reporting; and no other bias. Studies with a high risk of bias in any one or more key areas were considered to be at high risk of bias. Studies with a low risk of bias in all key areas were considered to have a low risk of bias. Otherwise, they were considered to have an unclear risk of bias.

### Data extraction

Two researchers (Wenfu Zhang and Yingting Wu) independently extracted data from eligible studies using predesigned data collection forms. The primary measures were changes in eGFR (1, 3, and 12 months postoperatively) and Scr, and the secondary measures were DGF, AR, graft loss, length of hospital stay and other periods (warm ischemic time, cold ischemic time and blocking time). In addition, the following information was extracted: first author's last name, year, country, donor type, patient demographics, hypertension, diabetes status, RIC type, RIC protocol, etc. Multiple forms of continuous data were converted to the mean and standard deviation. If the data were represented by medians and quartiles, we assumed a value of 1.35 standard deviations. If the data were reported as the median and range, we converted these values to estimate the mean and standard deviation. The mean standard deviation of the values at the same time point in other RCTs using the same intervention was used as the missing standard deviation. By discussing and resolving differences with a third researcher (Weidong Liang), a full consensus was finally reached across all projects. All raw data are listed in the attached table.

### Statistical analysis

Data were normalized to equivalent units prior to analysis. The results were evaluated as risk ratios (RRs) and mean differences (MDs) and their corresponding 95% confidence intervals (CIs) for dichotomous and continuous variables, respectively. Heterogeneity between eligible studies was assessed with the *Q* test and *I*^2^ test to assess the degree of between-study variability. We assessed the source of heterogeneity when *I*^2 ^> 50% or *P* < 0.05. If studies showed high heterogeneity, subgroup analyses or sensitivity analyses were performed on the results, excluding each trial involved in the pooled meta-analysis, one at a time, to see if the corresponding RR or MD changed significantly. Data synthesis was performed using a random-effects model or a fixed-effects model. A *P* value of <0.05 for the overall effect was considered statistically significant in all analyses. All statistical analyses were performed using Review Manager (version 5.3, Cochrane Collaboration, Oxford, UK).

## Results

### Search results and study characteristics

**Figure 1:**
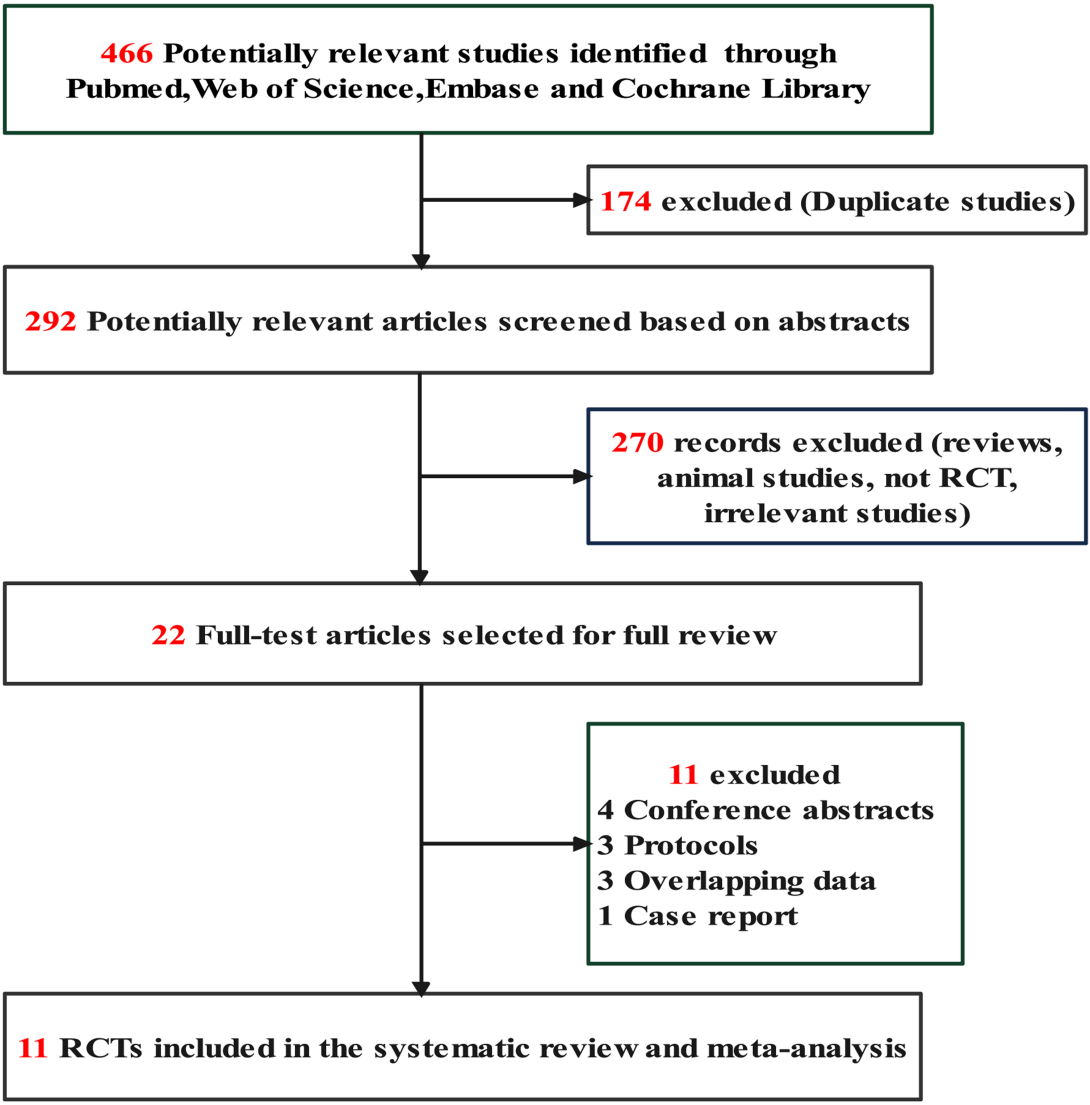
Flow chart of study selection. RCT, randomized controlled trial.

The study selection process is shown in Figure 1. A literature search yielded 466 potentially relevant records. By filtering titles, we removed 174 duplicate studies. After evaluating the abstract of each study, 270 studies were excluded because they did not meet the inclusion criteria. Subsequently, we carefully read the full text of the remaining 22 studies and excluded 11 studies: conference abstracts (*n* = 4), presentations (*n* = 1), overlapping data (*n* = 3) and protocols (*n* = 3). Finally, eleven RCTs were included in this systematic review and meta-analysis (6, 25–27, 29–32).

Eleven studies included a total of 1,145 patients with kidney transplantation or partial nephrectomy, of which 576 were randomly assigned to the RIC group and the remaining 569 to the control group. Of these studies, seven RCTs applied RIPC (6, 7, 25, 27, 29, 30, 33), three applied RIPeC (26, 28, 31), and the remaining one applied RIPoC (28). Eight studies were conducted on kidney transplantation (6, 26–29, 31–33), five of which involved living donors (6, 29, 31–33) and three of which involved deceased donors (26–28), and this meta-analysis focused on kidney transplantation. The remaining three studies concerned partial nephrectomy (25, 30, 33). Two studies investigated early and late ischemic preconditioning (7, 30), and in the current study, RIPC had two time windows, early RIPC and late RIPrC (34). In this meta-analysis, only early ischemic preconditioning was targeted.

In the included studies, the most common approach to inducing ischemia in RIC was by inflating a blood pressure cuff to a pressure of 200–250 mmHg for three or four cycles of 5 min with intervals of 5 min off, and only one study did not show the number of cycles. In terms of pressure, most studies set the blood-pressure cuff pressure at 200–250 mmHg, with two studies of blood-pressure cuff pressure reaching 300 mmHg, which may be associated with hypertension in patients involved. We think that because some of the patients included had hypertensive disease, the best method is to used ultrasound of a peripheral arterial pulse to confirm the absence of perfusion to a limb. This may be considered a “gold standard” for the presence of ischemia. In addition, In four studies, blood-pressure cuff were placed on the upper extremities and the rest on the upper thighs.

### Assessment of methodological quality

**Figure 2:**
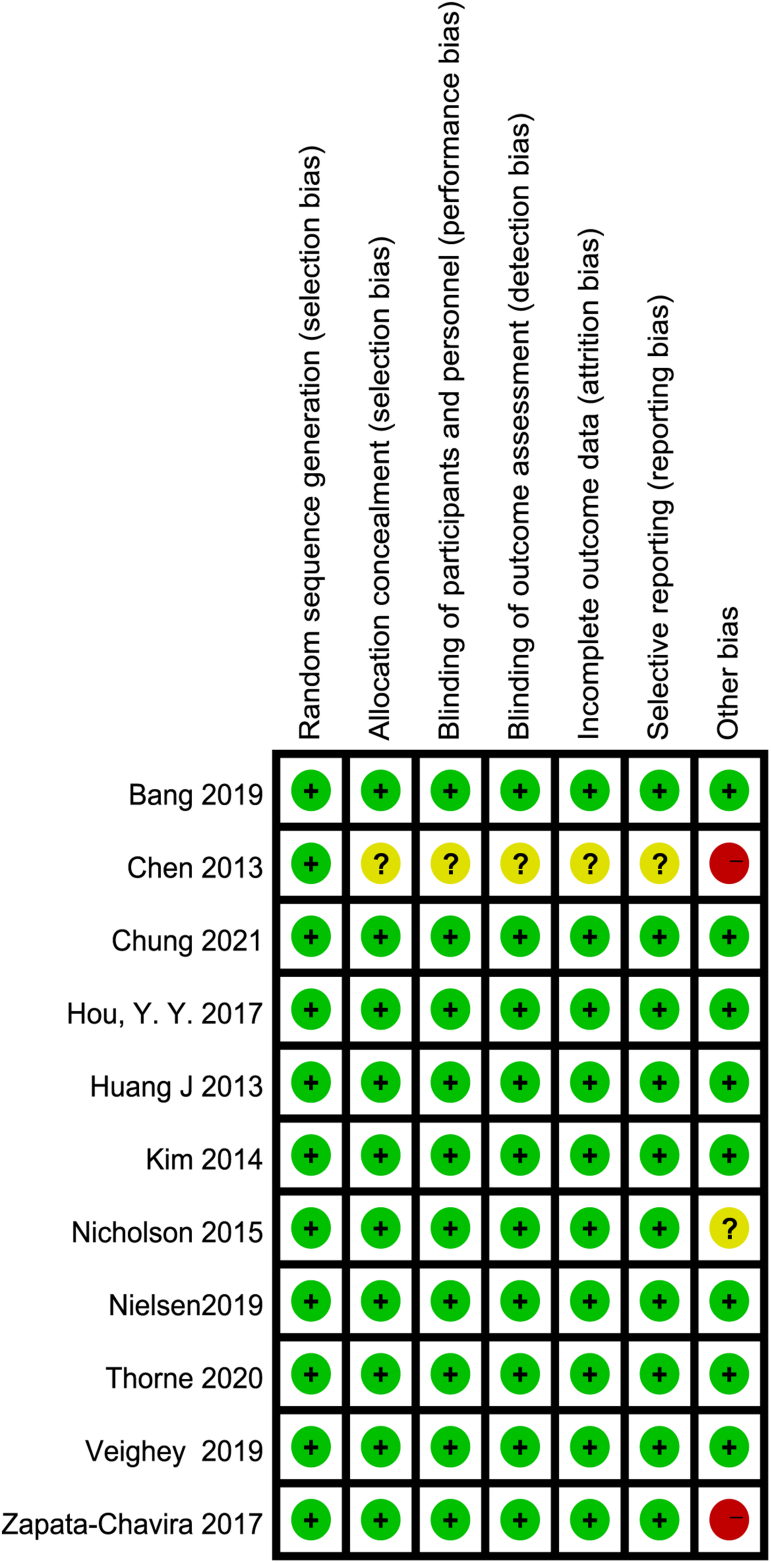
Summary of risk of bias: green: low risk of bias, yellow: unclear risk of bias, red: high risk of bias.

Details of the risk of bias are summarized in Figure 2. Eight studies were judged to be at low risk of bias (6, 7, 26–29, 32, 33), two were judged to be at high risk of bias due to small sample sizes (25, 31), and the remaining one was judged to have an unclear risk of bias (30). Ten reported appropriate allocation concealment and blinding (6, 7, 25–32). In the included studies, selective reporting was not found. Detailed quality assessments are summarized in Supplementary Table S1.

### Study outcomes

#### eGFR at 1 month, 3 months and 12 months

##### Kidney transplantation

**Figure 3:**
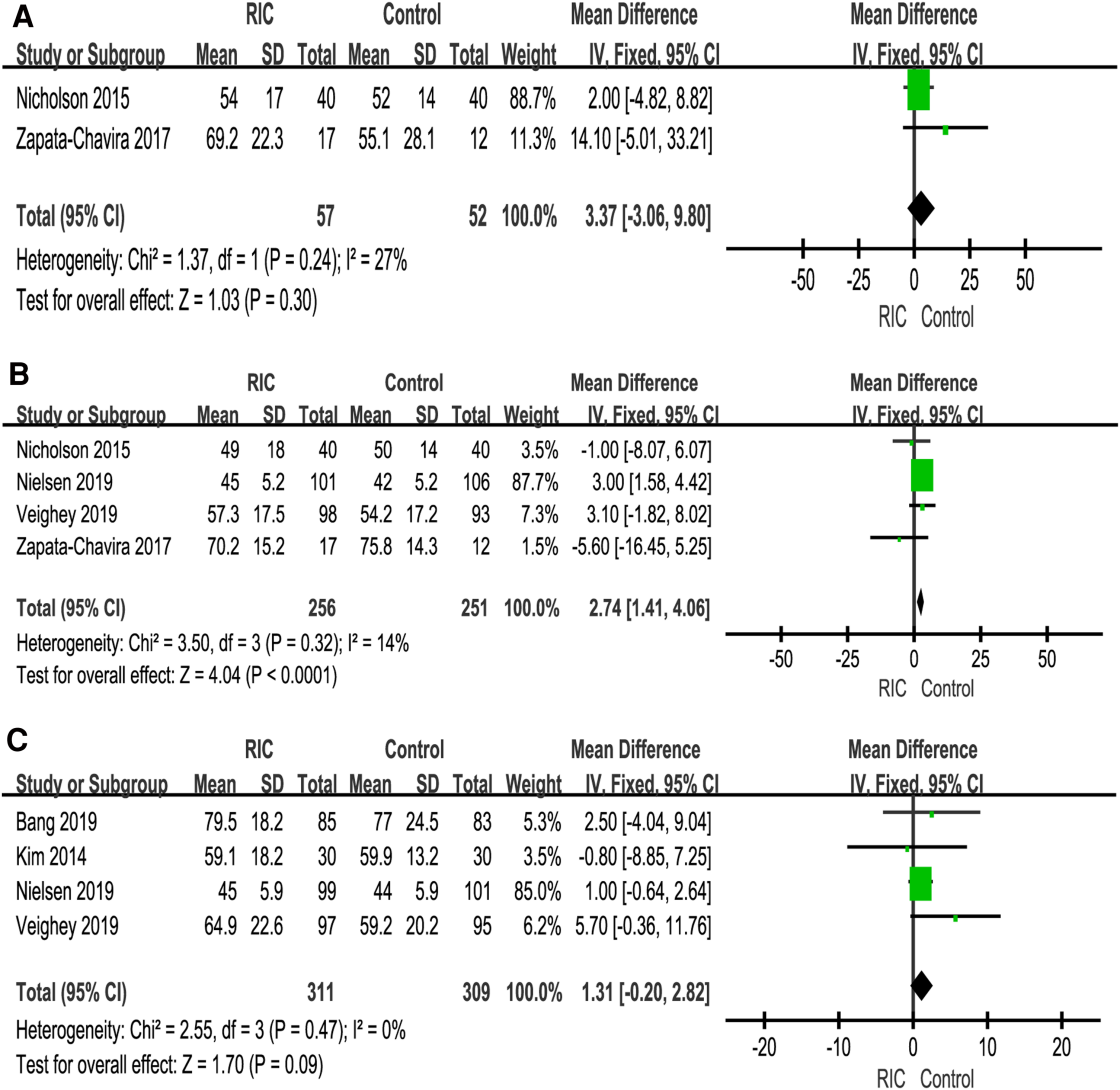
Forest plot with 95% confidence intervals for continuous primary end points. The eGFR at 1 month postoperation (**A**); eGFR at 3 months postoperation (**B**); eGFR at 12 months postoperation (**C**). RIC, remote ischemic conditioning; eGFR, estimated glomerular filtration rate.

Six studies reported eGFR, of which two studies reported the 1-month eGFR (27, 31), four studies reported the 3-month eGFR (6, 27, 30, 31), and four studies reported the 12-month eGFR (6, 26, 29, 30). No significant difference was observed between the two groups at 1- and 12 months (1-month RIC vs. control: *P *= 0.30; MD = 3.37, CI: −3.06 to 9.80, *I*^2 ^= 27% and 12 months RIC vs. control: *P *= 0.09; MD = 1.31, CI: −0.20 to 2.82, *I*^2 ^= 0, Figures 3A,C). There was a significant difference between the two groups at 3 months (RIC vs. control: *P *< 0.001; MD = 2.74, CI: 1.41 to 4.06; *I*^2 ^= 14%, Figure 3B).

##### Partial nephrectomy

Two studies reported eGFR on partial nephrectomy. Huang et al. (7) reported the first-month eGFR and sixth-month eGFR, but there was no significant difference in the values between the groups at the various time points. In addition, Hou et al. (28) reported the third-month eGFR, and there was a significant difference in the values between the groups (40.9 ± 6.6 ml/min with control vs. 45.7 ± 7.9 ml/min with RIC, *P* = 0.019, 95% CI for median difference 0.8–8.7).

#### Serum creatinine levels at different times

##### Kidney transplantation

**Figure 4:**
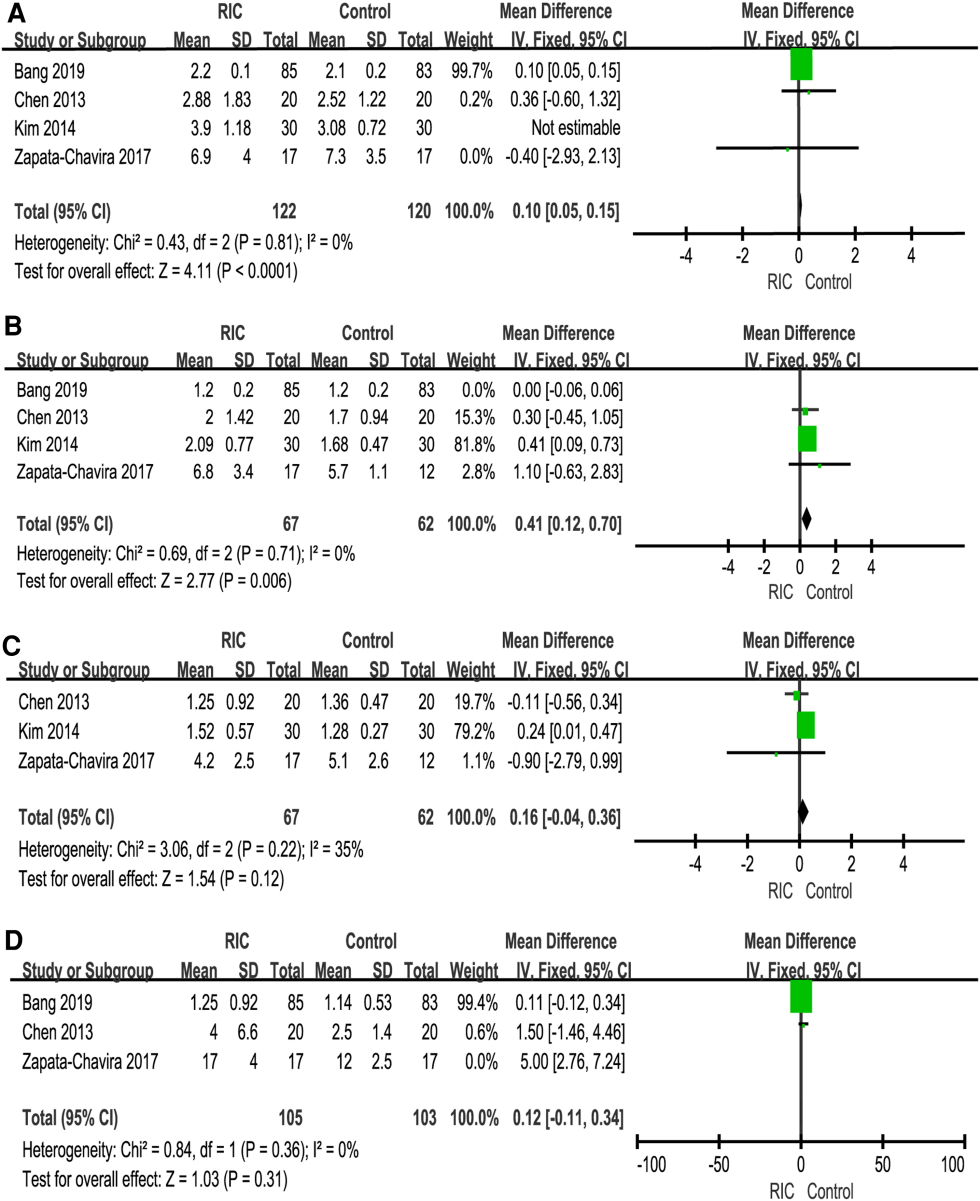
Forest plot with 95% confidence interval in serum creatinine (Scr), Scr levels at 1 day postoperatively (**A**), Scr levels at 2 days postoperatively (**B**), Scr levels at 3 days postoperatively (**C**), Scr levels at 7 days postoperatively (**D**). RIC, remote ischemic conditioning.

**Figure 5:**
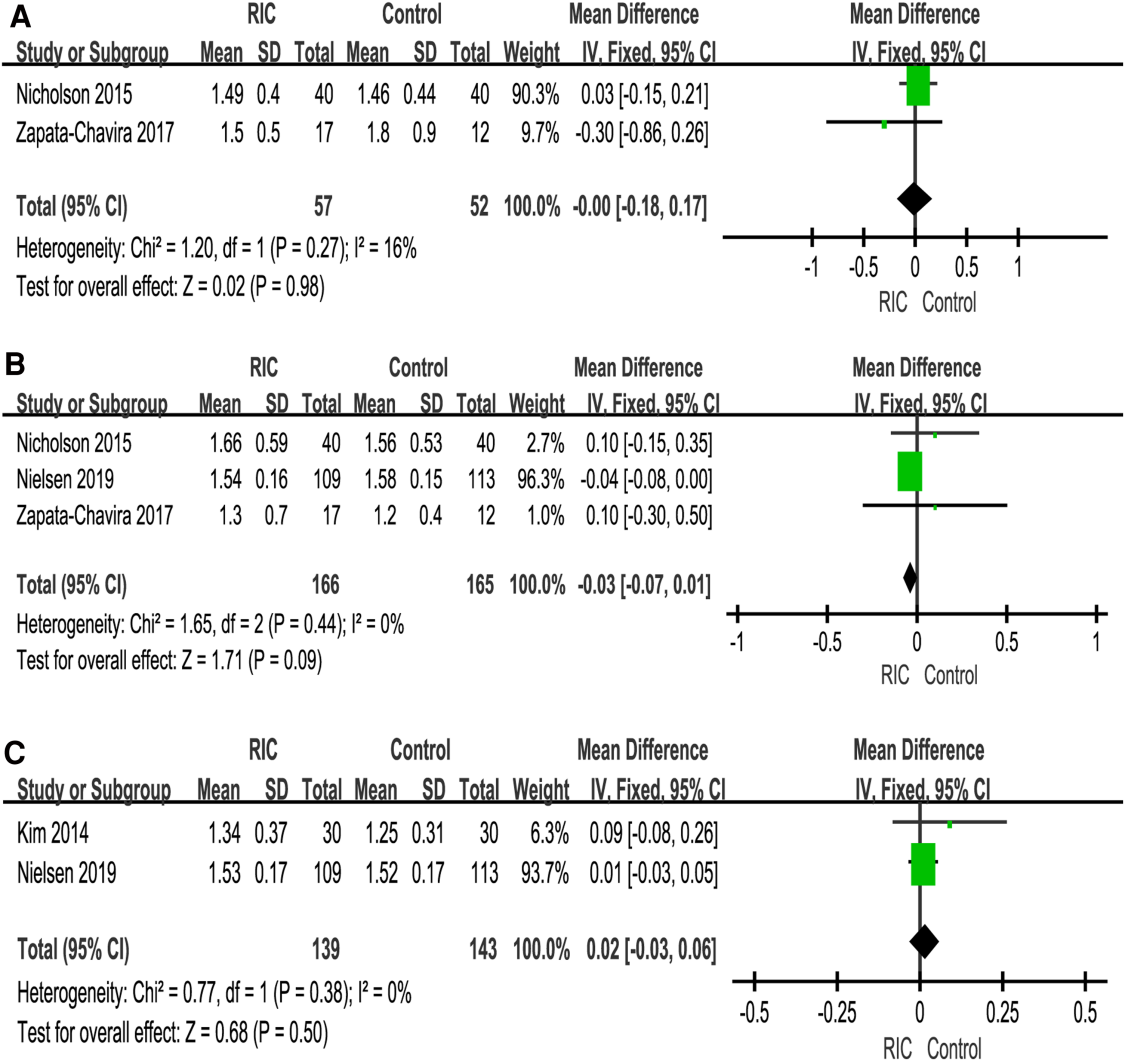
Forest plot with 95% confidence interval in serum creatinine (Scr), Scr levels at 1 month postoperatively (**A**), Scr levels at 3 months postoperatively (**B**), Scr levels at 12 months postoperatively (**C**). RIC, remote ischemic conditioning.

We reported serum creatinine levels at 1 day postoperatively, 2 days postoperatively, 3 days postoperatively, 7 days postoperatively, 1 month postoperatively, 3 months postoperatively and 12 months postoperatively. At 1 day postoperatively, the meta-analysis found high heterogeneity between the two groups. A subsequent sensitivity analysis was performed, and we found that after excluding one study (26), the heterogeneity was at the critical value, and there was a significant difference between the two groups (*P *< 0.001; MD = 0.10, CI: 0.05 to 0.15, *I*^2 ^= 0, Figure 4A). At 2 days postoperatively, we also found high heterogeneity between the two groups. After sensitivity analysis, it was found that the heterogeneity changed significantly, and there was a significant difference between the two groups (*P *= 0.006; MD = 0.41, CI: 0.12 to 0.70, *I*^2 ^= 0, Figure 4B). No heterogeneity or significant difference was found between the two groups at 3 days postoperatively (*P *= 0.61; MD = 0.09, CI: −0.25 to 0.42, *I*^2 ^= 35%, Figure 4C). At 7 days postoperatively, the meta-analysis found high heterogeneity between the two groups. A subsequent sensitivity analysis was performed, and we found that after excluding one study (31), the heterogeneity was at the critical value, but there was no significant difference between the two groups (*P *= 0.31; MD = 0.12, CI: −0.11 to 0.34, *I*^2 ^= 0, Figure 4D). In addition, no heterogeneity or significant difference was found between the two groups at 1 month postoperatively, 3 months postoperatively and 12 months postoperatively (Figure 5).

##### Partial nephrectomy

Levels of serum creatinine have been reported in two studies. Chung et al. (33) showed that there was no significant difference in the serum creatinine level between the two groups on the first postoperative day (median (interquartile range) 0.87 mg/dl (0.72–1.03) in the RIC group vs. 0.92 mg/dl (0.71–1.12) in the control group, *P* = 0.728). In addition, at 2 weeks after surgery, there was no significant difference in time-dependent changes in serum creatinine between the two groups. Huang et al. (7) showed that there was no significant difference in the serum creatinine level between the two groups at 1 month postoperatively (mean (SD) 0.81 mg/ml (0,28) in the RIC group vs. 0.81 mg/ml (0,19) in the control group, *P* = 0.933).

#### Incidence of DGF, AR and graft loss on kidney transplantation

**Figure 6:**
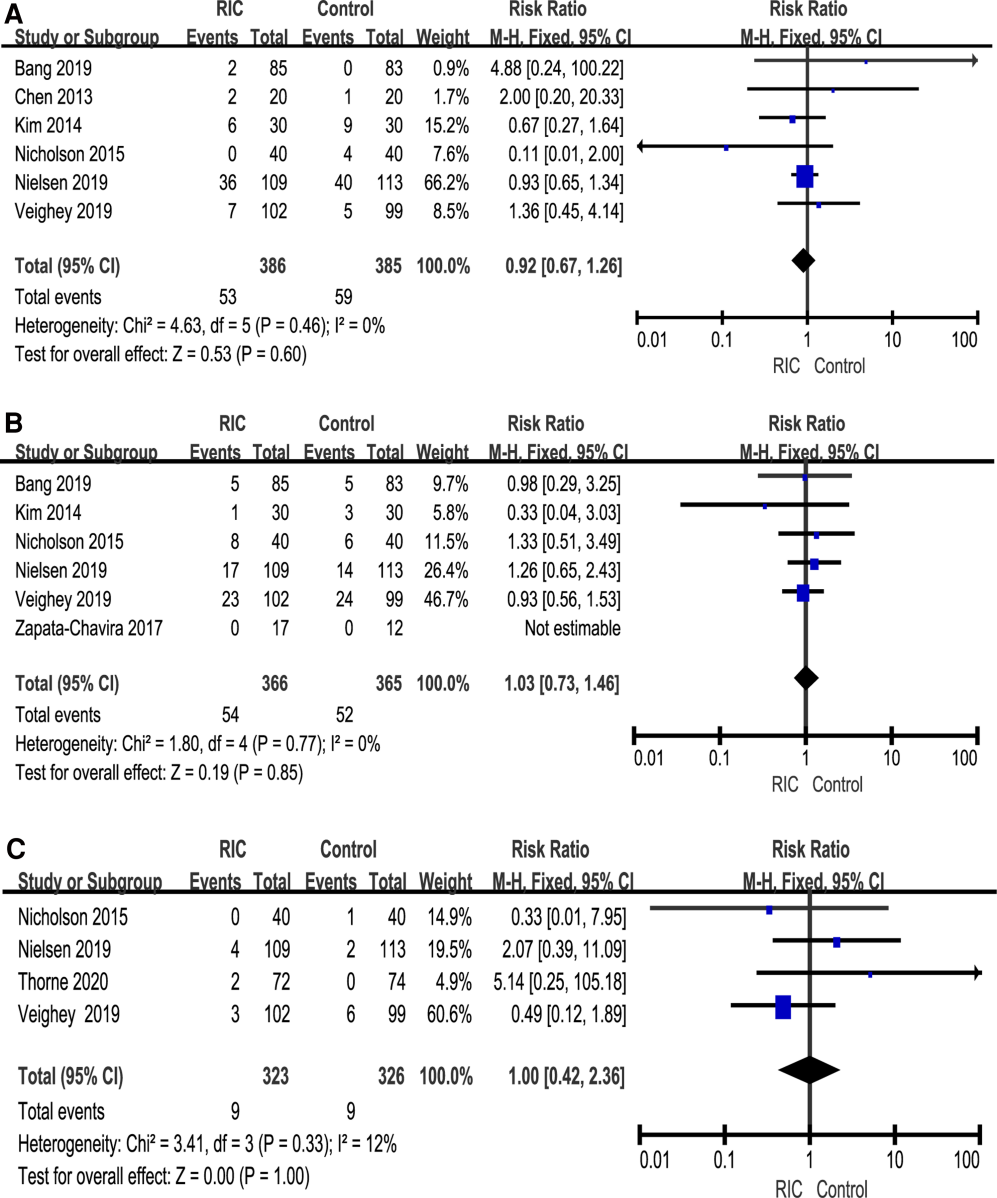
Forest plot with 95% confidence intervals for the rates of DGF (**A**), AR (**B**) and graft loss (**C**). RIC, remote ischemic conditioning; DGF, delayed graft function; AR, acute rejection.

The incidence of DGF was reported in six studies, and a meta-analysis of data from six trials was combined (6, 25–27, 29, 30). No heterogeneity was found between the two groups (*P *= 0.60; RR = 0.92, CI: 0.67 to 1.26, *I*^2 ^= 0, Figure 6A). Under the fixed effect model, the incidence of DGF in the RIC group showed a decreasing trend compared with that in the control group, but the decrease in the incidence of DGF was not statistically significant. Six studies reported the incidence of AR (6, 26, 27, 29–31), and meta-analysis found no significant difference between the RIC and control groups (*P *= 0.85; RR = 1.03, CI: 0.73 to 1.46, *I*^2 ^= 0, Figure 6B). Four studies reported the incidence of graft loss (6, 27, 30, 32), and the results showed no significant difference between the two (*P *= 1.00; RR = 1.00, CI: 0.42 to 2.36, *I*^2 ^= 12%, Figure 6C).

#### Length of hospital stay and other periods

##### Kidney transplantation

**Figure 7:**
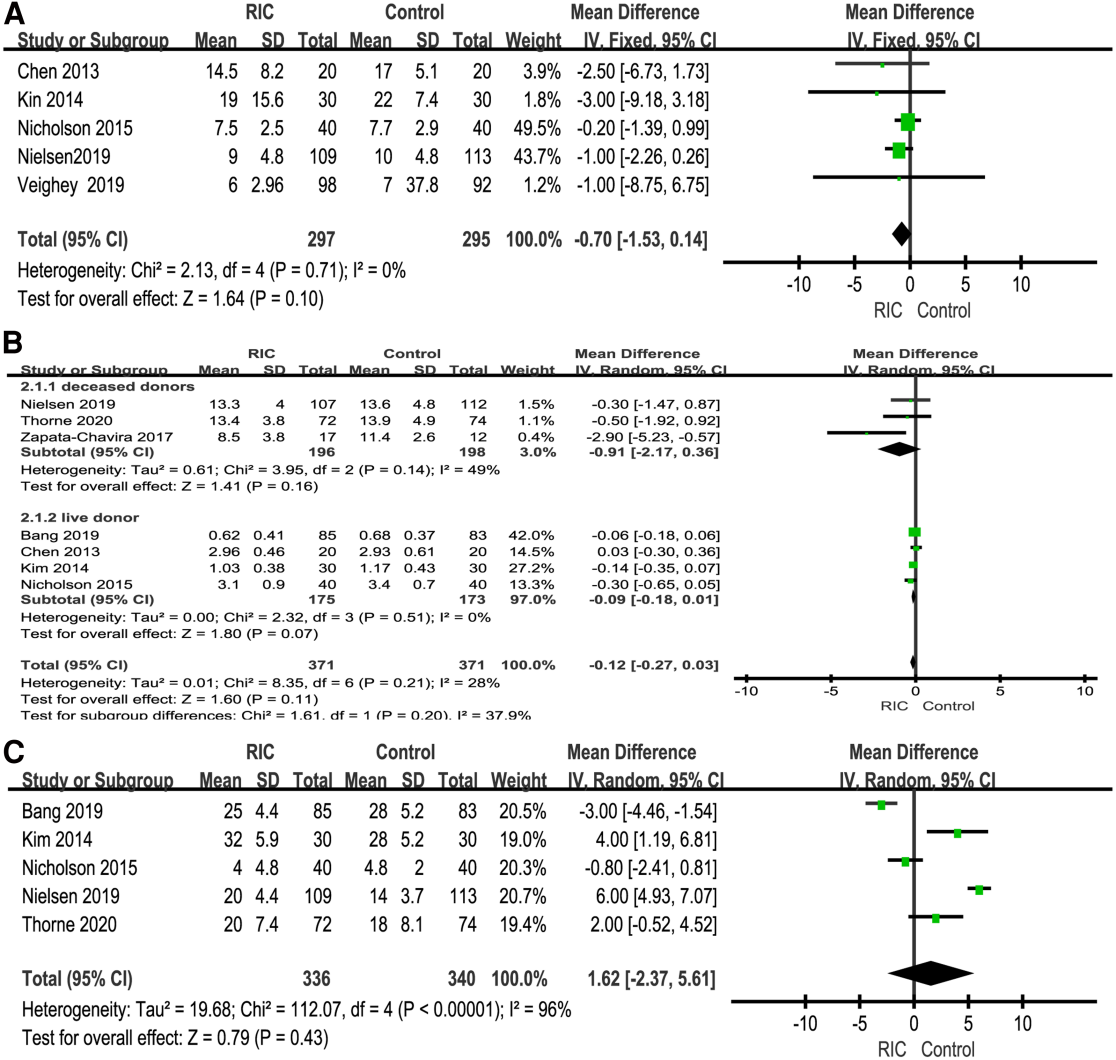
Forest plot with 95% confidence interval for length of hospital stay on kidney transplantation (**A**); meta-analysis of cold ischemic time (**B**) and warm ischemic time (**C**). RIC, remote ischemic conditioning.

A total of five studies reported the length of hospital stay (6, 25–27, 30). There was no significant difference in the length of hospital stay between the two groups (*P *= 0.10; MD = −0.70, CI: −1.53 to 0.14, *I*^2 ^= 0, Figure 7A). In addition, we also extracted cold ischemic time and warm ischemic time for merging, except that warm ischemic time showed significant heterogeneity (*P *= 0.43; MD = 1.62, CI: −2.37 to 5.61, *I*^2 ^= 96). The heterogeneity remained significant after sensitivity and subgroup analyses, suggesting that the results were unstable. None of the above results showed a significant difference between the two groups (Figure 7B, C).

##### Partial nephrectomy

**Figure 8:**
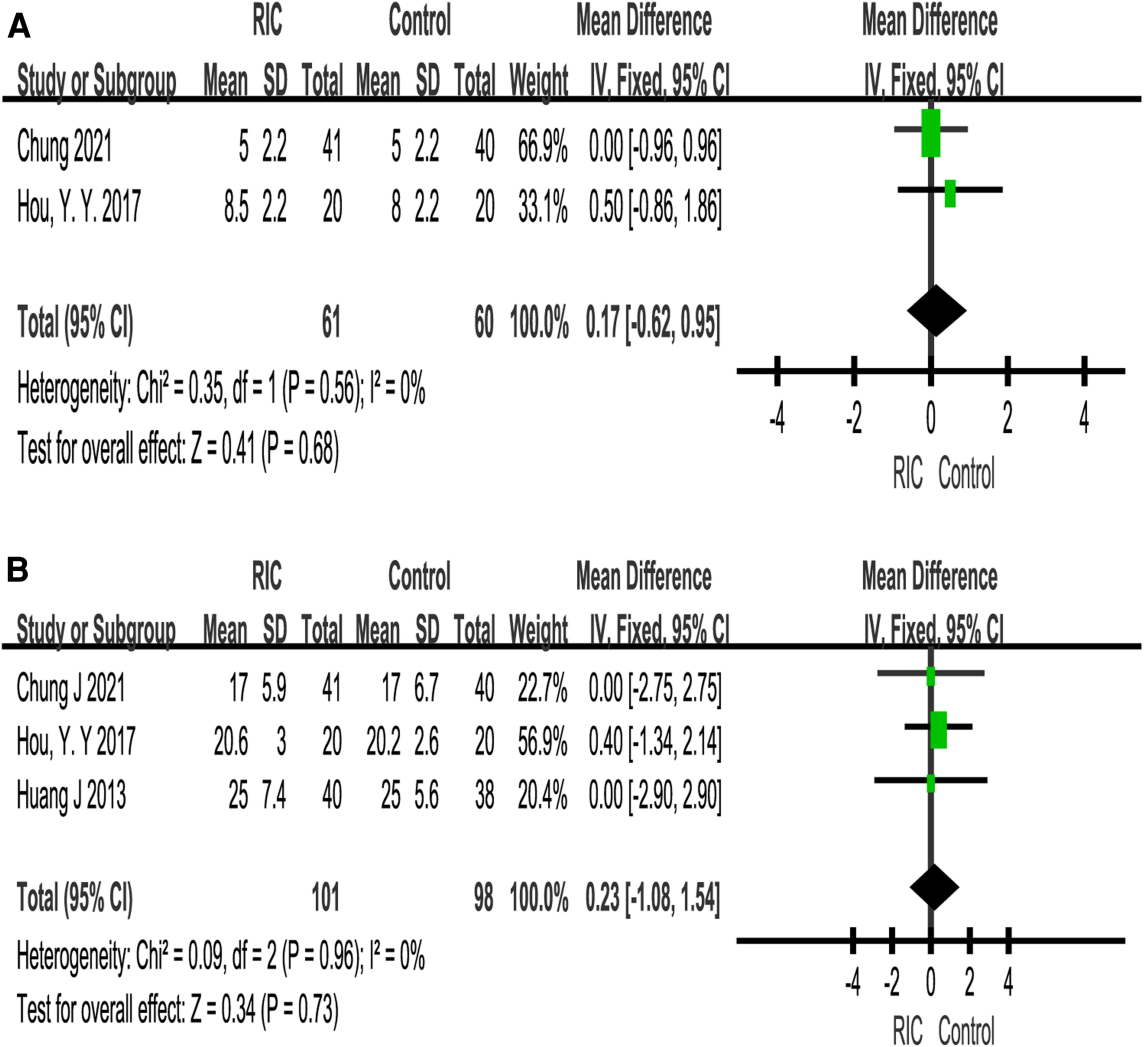
Forest plot with 95% confidence interval for length of hospital stay on partial nephrectomy (**A**); meta-analysis of cross-clamp time on partial nephrectomy (**B**). RIC, remote ischemic conditioning.

A total of two studies reported the length of hospital stay (28, 33). There was no significant difference in the length of hospital stay between the two groups (*P *= 0.68; MD = 0.17, CI: −0.62 to 0.95, *I*^2 ^= 0, Figure 8A). In terms of cross-clamp time, meta-analysis revealed that there was no significant difference between the two groups (*P *= 0.73; MD = 0.23, CI: −1.08 to 1.54, *I*^2 ^= 0, Figure 8B).

## Discussion

Our systematic review and meta-analysis included 11 RCTs. From the results, we found the following: (1) At the 3-month eGFR, patients receiving RIC intervention had significantly better outcomes than the control group; at the 1-month and the 12-month eGFR, although the two groups of patients’ eGFR did not show a significant difference, RIC slightly improved the patients’ eGFR. (2) RIC significantly reduced the serum creatinine level at 1 and 2 days postoperatively, but at other times, there was no significant difference between the two groups in Scr levels, and no nephroprotective effect of RIC was found. (3) In terms of DGF, AR, graft loss and length of hospital stay, there was no significant difference between the two groups. Although RIPC is easy to manipulate, the protective effect of RIPC in kidney transplantation surgery is controversial because it has only been shown to be based on rather short-term observations. RIC intervention seems to have no obvious effect on renal I/R injury.

RIC is a simple, inexpensive and noninvasive intervention to alleviate I/R injury (35), and RIPC has been rapidly applied since Murry et al. first proposed RIPC in canine cardiomyocytes in 1986 (36). In various clinical fields, this intervention has been applied in a variety of organs, has played a protective role and has been gradually employed from RIPC to RIPerC and RIPostC, all of which have achieved remarkable effects (17, 19, 32). However, the mechanism of RIC is still being explored and seems to be multimodal, possibly participating in protection through neural, humoral and systemic responses (10, 37). For example, RIC may promote the production of some active substances (opioids, adenosine, bradykinin and stromal cell-derived factor-1, etc.) and accelerate their entry into the blood, thus activating neural networks and acting on the target organs of the human body (38, 39). In addition, RIC may also activate antiapoptotic (40), antioedema (41), and anti-inflammatory signalling pathways (42), improve microvascular endothelial function (43), and affect the transcription of related genes and mitochondrial respiratory function (44, 45), thereby reducing the impact of I/R injury. Clinical trials on RIC renal protection have been conducted in a variety of surgical types, including cardiac surgery, macrovascular surgery, and percutaneous coronary intervention, and this approach has gradually become a promising renal protection strategy and has been demonstrated in experimental and clinical studies (46–48). At present, a large number of meta-analyses have shown the protective effect of RIC on the kidney, such as renal protection after coronary angiography or interventional patients with contrast agents (46). Meta-analyses conducted by Zhou et al. (49) show that the protective effect of RIC on kidney transplant patients needs more research to be elucidated. Systematic reviews performed by Farooqui et al. (50) show that RIPC can alleviate the ischemic-reperfusion injury of grafts in graft recipients.

During partial nephrectomy, the vessels in the renal pedicle must be temporarily clamped, resulting in I/R injury (51). The longer the time of heat ischemia, the greater the damage to renal function (52). Furthermore, during kidney transplantation, both the donor and the recipient experience renal I/R, which can result in acute or chronic renal failure due to I/R injury (27). Animal studies have shown (53) that RIPC has a protective effect against renal I/R injury and that this protective effect can reduce renal injury in both delayed and early RIPC regimens. However, Samadi et al. showed (20) that RIPC had little to no protection against renal I/R injury. Similarly, clinical studies have yielded disparate results. RIPC has been shown in some studies to protect renal I/R injury and improve renal function following living donor kidney transplantation (6, 29). However, Nicholson et al. (27) showed that RIC had no effect on renal function after living donor kidney transplantation. As a result, the protective effect of RIC on renal I/R injury has yet to be established. To investigate the protective effect of RIC on renal I/R injury, we conducted a systematic review and meta-analysis.

Our systematic review and meta-analysis included 11 studies involving 1,145 patients: 119 with partial nephrectomy and 1,026 with kidney transplantation. The results of this meta-analysis showed that RIC only decreased Scr at the first day and second days postoperatively, and RIC had no obvious effect on improving the eGFR and alleviating renal I/R injury. In this meta-analysis, the reasons why the RIC test results did not obtain a strong positive result may be as follows.

First, different RIC methods may have a certain impact on the results. In this experiment, seven studies involved RIPC, three studies involved RIPerC, and one study involved RIPostC. We tried to perform a subgroup analysis of RIC in different ways, but unfortunately, due to the limited studies included, we failed to obtain satisfactory results. It is known that RIC has three different application time points, RIPC, RIPerC and RIPostC, according to the different ischemic times of target organs (14). The most popular research is RIPC. RIPC is an intrinsic protective phenomenon in which 3–4 cycles of nonfatal regional ischemia followed by reperfusion to remote tissues protect vital organs including the brain, heart and kidney against sustained I/R-induced injury (17). Studies have shown that RIPC reduces the incidence of an early increase in Scr in patients with stable angina following elective percutaneous coronary intervention and may decrease the occurrence of acute kidney injury (AKI) in cardiovascular surgery patients (54, 55). RIPostC is a protective approach after I/R of target organs. RIPostC mainly focuses on brain protection, and most stroke patients cannot be predicted before stroke (56). Therefore, RIPostC is more effective after ischemia reperfusion injury. In animal experiments, RIPoC also has a certain renoprotective effect (57). However, it is rarely used in clinical practice. At present, there is no conclusion on the comparison of the three RIC modes, and it is not clear which RIC has a more obvious effect on renal protection. Therefore, more RCTs need to be conducted in future studies.

Second, different timings of RIC may have different effects on the results. One study compared early RIC (24 h before surgery) and late RIC (before surgery) and found that late RIC was more protective than early RIC (30). In addition, since the optimal number of cycles, pressure, duration, and cuff inflation site for RIC have not yet been determined, this is an important factor that cannot be ignored in the clinical application of RIC. Studies have shown that the most common site of RIC is the upper or lower extremities and that 3–4 cycles of I/R every 5 min will bring about beneficial effects (58). Studies have shown that the number of cycles and duration are not proportional to organ protection, and more cycles and longer duration do not provide better protection (9, 59). In terms of setting RIC pressure, studies have shown that most RIC pressures are approximately 200 mmHg or higher than 30–40 mmHg of systolic blood pressure due to differences in different populations and underlying diseases of patients (60). Due to the limited sample size of the included studies, it was not possible to analyse the current studies with different timings; consequently, it is crucial to increase the sample size in future studies.

Third, different patient populations, comorbidities, anaesthesia regimens, and surgical approaches can also affect outcomes. The donors for three kidney transplants in this study were deceased patients, which may have affected the stability of the results because deceased donors may have had a stronger inflammatory response (61). In addition, some studies have shown that PN can be divided into on-clamp PN and off-clamp PN. Off-clamp PN had negligible effects on renal function. Therefore, off-clamp PN is a safe surgical method with adequate tumor prognosis (52, 62, 63). However, in the articles we included, we failed to extract relevant data. Finally, patients had diabetes and hypertension among their comorbidities. Animal experiments have shown that diabetes can damage the PI3K-Akt signal transduction pathway, which plays a central role in intracellular pathways, in diabetic rat models (64). Therefore, diabetes may attenuate the myocardial protective effect of RIC.

Finally, our meta-analysis mainly analysed the outcomes of patients with kidney transplantation. In the included studies, most patients with kidney transplantation required hemodialysis before surgery, which led to repeated renal ischemia and reperfusion and may interfere with the effect of RIC. In addition, different from standardized animal experiments, clinical trials involve a variety of factors such as human leukocyte antigen mismatch and administration of immunosuppressive drugs, which may also interfere with the severity of I/R injury and hide the effects of RIC. In addition, in this meta-analysis, there may be systematic differences in the management of kidney transplant recipients with varying DGF durations between transplanting centres. It is known that the causative pathway resulting in DGF is often multifactorial, including immunological and nonimmunological factors that are present during organ procurement and immediately posttransplantation.

## Limitations

The above reasons may lead to the failure of our meta-analysis to obtain obvious positive results, but this study also has certain limitations. First, unlike previous meta-analyses on nephroprotective effects, our meta-analyses focused only on patients with kidney transplantation. Due to the small number of studies, we did not perform subgroup analyses for the primary outcome. Second, the included studies also had different ischemic manipulation methods (ischemia time and reperfusion time) and different interventions (RIC approach). It is not clear whether the differences between RIC methods affect the effectiveness of renal I/R injury, which should be verified by more RCTs. In addition, some of the included studies did not provide the primary outcome we needed and required small sample sizes, which led to a certain risk of bias. In a recent meta-analysis, RIC was found to reduce the risk of AKI in organs such as the heart (65). Of the included studies, only one reported the incidence of AKI (25). In addition, serum biomarkers such as cystatin C and neutrophil gelatinase-associated lipoprotein can reflect renal function to a certain extent (30). Therefore, follow-up studies can further focus on the incidence of AKI and certain biomarkers to better evaluate the superiority of RIC in renal surgery.

## Conclusion

Remote ischemia conditioning on reducing Scr and improving eGFR seemed to be very weak, and we did not observe a significant protective effect of RIC on renal I/R injury. Due to small sample sizes, more studies and meta-analyses using stricter inclusion criteria are needed to elucidate the nephroprotective effect of RIC in renal surgery in the future.

## Data Availability

The datasets presented in this study can be found in online repositories. The names of the repository/repositories and accession number(s) can be found in the article/**Supplementary Material**.

## References

[1] Piper HM García-Dorado D Ovize M. A fresh look at reperfusion injury. Cardiovasc Res. (1998) 38:291–300. 10.1016/s0008-6363(98)00033-9 9709390

[2] Robertson FP Magill LJ Wright GP Fuller B Davidson BR. A systematic review and meta-analysis of donor ischaemic preconditioning in liver transplantation. Transpl Int. (2016) 29:1147–54. 10.1111/tri.12849 27564598

[3] Yuan Y Guo Q Ye Z Pingping X Wang N Song Z. Ischemic postconditioning protects brain from ischemia/reperfusion injury by attenuating endoplasmic reticulum stress-induced apoptosis through PI3K-akt pathway. Brain Res. (2011) 1367:85–93. 10.1016/j.brainres.2010.10.017 20940001

[4] Verdouw PD Gho BC Koning MM Schoemaker RG Duncker DJ. Cardioprotection by ischemic and nonischemic myocardial stress and ischemia in remote organs. Implications for the concept of ischemic preconditioning. Ann N Y Acad Sci. (1996) 793:27–42. 10.1111/j.1749-6632.1996.tb33502.x 8906153

[5] Poston JT Koyner JL. Sepsis associated acute kidney injury. BMJ. (2019) 364:k4891. 10.1136/bmj.k4891 30626586 PMC6890472

[6] Veighey KV Nicholas JM Clayton T Knight R Robertson S Dalton N Early remote ischaemic preconditioning leads to sustained improvement in allograft function after live donor kidney transplantation: long-term outcomes in the REnal protection against ischaemia–reperfusion in transplantation (REPAIR) randomised trial. Br J Anaesth. (2019) 123:584–91. 10.1016/j.bja.2019.07.019 31521337

[7] Huang J Chen Y Dong B Kong W Zhang J Xue W Effect of remote ischaemic preconditioning on renal protection in patients undergoing laparoscopic partial nephrectomy: a ‘blinded’ randomised controlled trial. BJU Int. (2013) 112:74–80. 10.1111/bju.12004 23452148

[8] Tang C Hu Y Gao J Jiang J Shi S Wang J Dexmedetomidine pretreatment attenuates myocardial ischemia reperfusion induced acute kidney injury and endoplasmic reticulum stress in human and rat. Life Sci. (2020) 257:118004. 10.1016/j.lfs.2020.118004 32621918

[9] Heusch G Bøtker HE Przyklenk K Redington A Yellon D. Remote ischemic conditioning. J Am Coll Cardiol. (2015) 65:177–95. 10.1016/j.jacc.2014.10.031 25593060 PMC4297315

[10] Costa FLD Teixeira RKC Yamaki VN Valente AL Silva AMF Brito MVH Remote ischemic conditioning temporarily improves antioxidant defense. J Surg Res. (2016) 200:105–9. 10.1016/j.jss.2015.07.031 26316445

[11] Hunter JP Hosgood SA Barlow AD Nicholson ML. Ischaemic conditioning reduces kidney injury in an experimental large-animal model of warm renal ischaemia. Br J Surg. (2015) 102:1517–25. 10.1002/bjs.9909 26263908

[12] Kocman EA Ozatik O Sahin A Guney T Kose AA Dag I Effects of ischemic preconditioning protocols on skeletal muscle ischemia-reperfusion injury. J Surg Res. (2015) 193:942–52. 10.1016/j.jss.2014.09.032 25438960

[13] Chen G Thakkar M Robinson C Doré S. Limb remote ischemic conditioning: mechanisms, anesthetics, and the potential for expanding therapeutic options. Front Neurol. (2018) 9:40. 10.3389/fneur.2018.00040 29467715 PMC5808199

[14] Bromage DI Pickard JM Rossello X Ziff OJ Burke N Yellon DM Remote ischaemic conditioning reduces infarct size in animal in vivo models of ischaemia-reperfusion injury: a systematic review and meta-analysis. Cardiovasc Res. (2017) 113:288–97. 10.1093/cvr/cvw219 28028069 PMC5408955

[15] Li DY Shi XJ Li W Sun XD Wang GY. Ischemic preconditioning and remote ischemic preconditioning provide combined protective effect against ischemia/reperfusion injury. Life Sci. (2016) 150:76–80. 10.1016/j.lfs.2016.02.077 26920632

[16] Zong Y Jiang L Zhang M Zhou F Qi W Li S Limb remote ischemic postconditioning protects cerebral ischemia from injury associated with expression of HIF-1alpha in rats. BMC Neurosci. (2015) 16:97. 10.1186/s12868-015-0235-6 26715469 PMC4696280

[17] Heusch G. Molecular basis of cardioprotection: signal transduction in ischemic pre-, post-, and remote conditioning. Circ Res. (2015) 116:674–99. 10.1161/CIRCRESAHA.116.305348 25677517

[18] Hausenloy DJ Candilio L Evans R Ariti C Jenkins DP Kolvekar S Remote ischemic preconditioning and outcomes of cardiac surgery. N Engl J Med. (2015) 373:1408–17. 10.1056/NEJMoa1413534 26436207

[19] Wu G Chen M Wang X Kong E Yu W Sun Y Effect of remote ischemic preconditioning on hepatic ischemia-reperfusion injury in patients undergoing liver resection: a randomized controlled trial. Minerva Anestesiol. (2020) 86:252–60. 10.23736/S0375-9393.19.13838-2 31808659

[20] Samadi M Tabibian F Moradzadeh K Nassiri SM Gheisari Y. Evaluating the effect of remote ischemic preconditioning on kidney ischemia-reperfusion injury. J Res Med Sci. (2020) 25:6. 10.4103/jrms.JRMS_249_19 32055246 PMC7003542

[21] Pan T Jia P Chen N Fang Y Liang Y Guo M Delayed remote ischemic preconditioning ConfersRenoprotection against septic acute kidney injury via exosomal miR-21. Theranostics. (2019) 9:405–23. 10.7150/thno.29832 30809283 PMC6376188

[22] Oral K Akan M Özkardeşler S Boztaş N Ergür BU Güneli ME Comparison of direct and remote ischaemic preconditioning of renal ischaemia reperfusion injury in rats. Turk J Anaesthesiol Reanim. (2018) 46:453–61. 10.5152/TJAR.2018.07992 30505608 PMC6223872

[23] Moher D Liberati A Tetzlaff J Altman DG, PRISMA Group. Preferred reporting items for systematic reviews and meta-analyses: the PRISMA statement. PLoS Med. (2009) 6:e1000097. 10.1371/journal.pmed.1000097 19621072 PMC2707599

[24] Higgins JP Altman DG Gøtzsche PC Jüni P Moher D Oxman AD The cochrane collaboration's tool for assessing risk of bias in randomised trials. BMJ. (2011) 343:d5928. 10.1136/bmj.d5928 22008217 PMC3196245

[25] Chen Y Zheng H Wang X Zhou Z Luo A Tian Y. Remote ischemic preconditioning fails to improve early renal function of patients undergoing living-donor renal transplantation: a randomized controlled trial. Transplantation. (2013) 95:e2–4. 10.1097/TP.0b013e3182782f3a 23325011

[26] Kim WH Lee JH Ko JS Min JJ Gwak MS Kim GS Effect of remote ischemic postconditioning on patients undergoing living donor liver transplantation. Liver Transpl. (2014) 20:1383–92. 10.1002/lt.23960 25046844

[27] Nicholson ML Pattenden CJ Barlow AD Hunter JP Lee G Hosgood SA. A double blind randomized clinical trial of remote ischemic conditioning in live donor renal transplantation. Medicine. (2015) 94:e1316. 10.1097/MD.0000000000001316 26252316 PMC4616604

[28] Hou YY Li Y He SF Song J Yu DX Wong GTC Effects of differential-phase remote ischemic preconditioning intervention in laparoscopic partial nephrectomy: a single blinded, randomized controlled trial in a parallel group design. J Clin Anesth. (2017) 41:21–8. 10.1016/j.jclinane.2017.05.017 28802596

[29] Bang JY Kim SG Oh J Kim SO Go YJ Hwang GS Impact of remote ischemic preconditioning conducted in living kidney donors on renal function in donors and recipients following living donor kidney transplantation: a randomized clinical trial. J Clin Med. (2019) 8(5);713. 10.3390/jcm8050713 PMC6572316 31137470

[30] Nielsen MB Krogstrup NV Oltean M Nieuwenhuijs-Moeke GJ Dor F Birn H Remote ischaemic conditioning and early changes in plasma creatinine as markers of one year kidney graft function-a follow-up of the CONTEXT study. PloS One. (2019) 14:e0226882. 10.1371/journal.pone.0226882 31887168 PMC6936785

[31] Zapata-Chavira H Hernandez-Guedea M Jimenez-Perez JC Perez-Rodriguez E Munoz-Espinosa L Munoz-Maldonado G Modulation of remote ischemic preconditioning by proinflammatory cytokines in renal transplant recipients. J Invest Surg. (2019) 32:63–71. 10.1080/08941939.2017.1375052 29083941

[32] Thorne AM Huang H O'Brien DP Eijken M Krogstrup NV Norregaard R Subclinical effects of remote ischaemic conditioning in human kidney transplants revealed by quantitative proteomics. Clin Proteomics. (2020) 17:39. 10.1186/s12014-020-09301-x 33292164 PMC7607690

[33] Chung J Hur M Cho H Bae J Yoon HK Lee HJ The effect of remote ischemic preconditioning on serum creatinine in patients undergoing partial nephrectomy: a randomized controlled trial. J Clin Med. (2021) 10(8);1636. 10.3390/jcm10081636 PMC8069991 33921503

[34] Tapuria N Kumar Y Habib MM Abu Amara M Seifalian AM Davidson BR. Remote ischemic preconditioning: a novel protective method from ischemia reperfusion injury–a review. J Surg Res. (2008) 150:304–30. 10.1016/j.jss.2007.12.747 19040966

[35] Liu A Yang B. Roles of TRPM7 in renal ischemia-reperfusion injury. Curr Protein Pept Sci. (2019) 20:777–88. 10.2174/1389203720666190507102948 31060485

[36] Murry CE Jennings RB Reimer KA. Preconditioning with ischemia: a delay of lethal cell injury in ischemic myocardium. Circulation. (1986) 74:1124–36. 10.1161/01.cir.74.5.1124 3769170

[37] Liang W Lin C Yuan L Chen L Guo P Li P Preactivation of Notch1 in remote ischemic preconditioning reduces cerebral ischemia-reperfusion injury through crosstalk with the NF-κB pathway. J Neuroinflammation. (2019) 16:181. 10.1186/s12974-019-1570-9 31526384 PMC6747758

[38] Belon AR Tannuri ACA de Albuquerque Rangel Moreira D Figueiredo JL da Silva AM Serafini S Impact of three methods of ischemic preconditioning on ischemia-reperfusion injury in a pig model of liver transplantation. J Invest Surg. (2022) 35(4):900–9. 10.1080/08941939.2021.1933274 34180750

[39] Xue YJ Chen SN Chen WG Wu GQ Liao YF Xu JB Cripto-1 expression in patients with clear cell renal cell carcinoma is associated with poor disease outcome. J Exp Clin Cancer Res. (2019) 38:378. 10.1186/s13046-019-1386-6 31455359 PMC6712621

[40] Lv J Guan W You Q Deng L Zhu Y Guo K RIPC provides neuroprotection against ischemic stroke by suppressing apoptosis via the mitochondrial pathway. Sci Rep. (2020) 10:5361. 10.1038/s41598-020-62336-w 32210331 PMC7093414

[41] Li S Hu X Zhang M Zhou F Lin N Xia Q Remote ischemic post-conditioning improves neurological function by AQP4 down-regulation in astrocytes. Behav Brain Res. (2015) 289:1–8. 10.1016/j.bbr.2015.04.024 25907740

[42] Liu C Yang J Zhang C Geng X Zhao H. Remote ischemic conditioning reduced cerebral ischemic injury by modulating inflammatory responses and ERK activity in type 2 diabetic mice. Neurochem Int. (2020) 135:104690. 10.1016/j.neuint.2020.104690 31981607

[43] Rytter N Carter H Piil P Sorensen H Ehlers T Holmegaard F Ischemic preconditioning improves microvascular endothelial function in remote vasculature by enhanced prostacyclin production. J Am Heart Assoc. (2020) 9:e016017. 10.1161/JAHA.120.016017 32750305 PMC7792245

[44] Hu S Dong H Zhang H Wang S Hou L Chen S Noninvasive limb remote ischemic preconditioning contributes neuroprotective effects via activation of adenosine A1 receptor and redox status after transient focal cerebral ischemia in rats. Brain Res. (2012) 1459:81–90. 10.1016/j.brainres.2012.04.017 22560096

[45] Han F Wan S Sun Q Chen N Li H Zheng L Donor plasma mitochondrial DNA is correlated with posttransplant renal allograft function. Transplantation. (2019) 103:2347–58. 10.1097/TP.0000000000002598 30747854

[46] Zhan B Zhu B Hu J Huang Q Bao H Huang X The efficacy of remote ischemic conditioning in preventing contrast-induced nephropathy among patients undergoing coronary angiography or intervention: an updated systematic review and meta-analysis. Ann Noninvasive Electrocardiol. (2020) 25:e12706. 10.1111/anec.12706 31605431 PMC7358796

[47] Pranata R Tondas AE Vania R Toruan MPL Lukito AA Siswanto BB. Remote ischemic preconditioning reduces the incidence of contrast-induced nephropathy in patients undergoing coronary angiography/intervention: systematic review and meta-analysis of randomized controlled trials. Catheter Cardiovasc Interv. (2020) 96:1200–12. 10.1002/ccd.28709 31912996

[48] Haller PM Vargas KG Haller MC Piackova E Wojta J Gyöngyösi M Remote ischaemic conditioning for myocardial infarction or elective PCI: systematic review and meta-analyses of randomised trials. European Heart Journal Acute Cardiovascular Care. (2020) 9:82–92. 10.1177/2048872618784150 29911392

[49] Zhou CC Ge YZ Yao WT Wu R Xin H Lu TZ Limited clinical utility of remote ischemic conditioning in renal transplantation: a meta-analysis of randomized controlled trials. PLoS One. (2017) 12:e0170729. 10.1371/journal.pone.0170729 28129389 PMC5271340

[50] Farooqui W Pommergaard HC Rasmussen A. Remote ischemic preconditioning of transplant recipients to reduce graft ischemia and reperfusion injuries: a systematic review. Transplant Rev. (2018) 32:10–5. 10.1016/j.trre.2017.06.001 28637593

[51] Chae MS Shim JW Choi H Hong SH Lee JY Jeong W Effects of multimodal bundle with remote ischemic preconditioning and intrathecal analgesia on early recovery of estimated glomerular filtration rate after robot-assisted laparoscopic partial nephrectomy for renal cell carcinoma. Cancers. (2022) 14(8);1985. 10.3390/cancers14081985 PMC9032668 35454891

[52] Simone G Misuraca L Tuderti G Minisola F Ferriero M Romeo G Purely off-clamp robotic partial nephrectomy: preliminary 3-year oncological and functional outcomes. Int J Urol. (2018) 25:606–14. 10.1111/iju.13580 29663528

[53] Varga G Ghanem S Szabo B Nagy K Pal N Tanczos B Which remote ischemic preconditioning protocol is favorable in renal ischemia-reperfusion injury in the rat? Clin Hemorheol Microcirc. (2020) 76:439–51. 10.3233/CH-200916 32804120

[54] Wojciechowska M Zarębiński M Pawluczuk P Gralak-Łachowska D Pawłowski L Loska W Remote ischemic preconditioning in renal protection during elective percutaneous coronary intervention. Adv Exp Med Biol. (2018) 1116:19–25. 10.1007/5584_2018_282 30267308

[55] Zhou H Yang L Wang G Zhang C Fang Z Lei G Remote ischemic preconditioning prevents postoperative acute kidney injury after open total aortic arch replacement: a double-blind, randomized, sham-controlled trial. Anesth Analg. (2019) 129:287–93. 10.1213/ANE.0000000000004127 30896603

[56] Yu HH Ma XT Ma X Chen M Chu YH Wu LJ Remote limb ischemic postconditioning protects against ischemic stroke by promoting regulatory T cells thriving. J Am Heart Assoc. (2021) 10:e023077. 10.1161/JAHA.121.023077 34726065 PMC8751947

[57] Han D Wang J Wen L Sun M Liu H Gao Y. Remote limb ischemic postconditioning protects against ischemic stroke via modulating microglia/macrophage polarization in mice. J Immunol Res. (2021) 2021:6688053. 10.1155/2021/6688053 33688509 PMC7910075

[58] Vinten-Johansen J Shi W. Perconditioning and postconditioning: current knowledge, knowledge gaps, barriers to adoption, and future directions. J Cardiovasc Pharmacol Ther. (2011) 16:260–6. 10.1177/1074248411415270 21821526

[59] Giannopoulos G Vrachatis DA Panagopoulou V Vavuranakis M Cleman MW Deftereos S. Remote ischemic conditioning and renal protection. J Cardiovasc Pharmacol Ther. (2017) 22:321–9. 10.1177/1074248417702480 28443376

[60] Cho YJ Kim WH. Perioperative cardioprotection by remote ischemic conditioning. Int J Mol Sci. (2019) 20(19);4839. 10.3390/ijms20194839 31569468 PMC6801656

[61] Wu J Feng X Huang H Shou Z Zhang X Wang R Remote ischemic conditioning enhanced the early recovery of renal function in recipients after kidney transplantation: a randomized controlled trial. J Surg Res. (2014) 188:303–8. 10.1016/j.jss.2013.06.058 24556231

[62] Simone G Capitanio U Tuderti G Presicce F Leonardo C Ferriero M On-clamp versus off-clamp partial nephrectomy: propensity score-matched comparison of long-term functional outcomes. Int J Urol. (2019) 26:985–91. 10.1111/iju.14079 31342589

[63] Simone G Tuderti G Anceschi U Papalia R Ferriero M Misuraca L Oncological outcomes of minimally invasive partial versus minimally invasive radical nephrectomy for cT1-2/N0/M0 clear cell renal cell carcinoma: a propensity score-matched analysis. World J Urol. (2017) 35:789–94. 10.1007/s00345-016-1923-27578234

[64] Ren BC Zhang YF Liu SS Cheng XJ Yang X Cui XG Curcumin alleviates oxidative stress and inhibits apoptosis in diabetic cardiomyopathy via Sirt1-Foxo1 and PI3K-akt signalling pathways. J Cell Mol Med. (2020) 24:12355–67. 10.1111/jcmm.15725 32961025 PMC7687015

[65] Li B Lang X Cao L Wang Y Lu Y Feng S Effect of remote ischemic preconditioning on postoperative acute kidney injury among patients undergoing cardiac and vascular interventions: a meta-analysis. J Nephrol. (2017) 30:19–33. 10.1007/s40620-016-0301-x 27091767 PMC5316401

